# Intellectual Disability in Two Brothers Caused by De Novo Novel Unbalanced Translocation (13;18) (q34,q23) and De Novo Microdeletion 6q25 Syndrome

**DOI:** 10.7759/cureus.6778

**Published:** 2020-01-26

**Authors:** Amal M Alhashem, Manal S Almohaid, Lina Alanazi, Hedayah Alhabardi

**Affiliations:** 1 Pediatrics / Medical Genetics, Prince Sultan Medical Military City, Riyadh, SAU; 2 Pediatrics, Princess Nourah Bint Abdulrahman University, Riyadh, SAU

**Keywords:** 18q23 microduplication, fg syndrome, intellectual disability, multiple congenital anomalies, hypotonia, developmental/speech delay, autism, 13q34 microdeletion, 6q25 deletion

## Abstract

We report here two brothers with an intellectual disability (ID), dysmorphic features, speech delay, and congenital hypotonia, with chromosomal microarray confirmed. However, two different de novo chromosomal aberrations; unbalanced translocations (13;18) (q34,q23) were found in the elder boys and de novo 6q25 deletion in the second boy. The boy with 13q34 microdeletion and 18q23 microduplication suffered from ID, obesity, dysmorphic features, speech delay, and seizure while the one with 6q25 deletion presented with ID and speech delay. Both parents were tested and were normal. The third child had mild hypotonia at infancy, which improved later. Whole-exome sequencing (WES) showed the three boys carried a likely benign variant in MED12, inherited from the healthy, asymptomatic mother. The father suffered from rheumatoid arthritis and was on chemotherapy during the conception of the first two affected boys. This report places emphasis on the use of a chromosomal microarray in patients with ID, even with familial cases, and reports the paternal use of methotrexate.

## Introduction

The alteration of gene dosage due to gains or deletions of large genomic regions causes many genetic disorders that are frequently associated with intellectual disability (ID), multiple congenital anomalies (MCA), autistic spectrum disorders (ASD), and other phenotypic findings [1].

Many microdeletion/duplications syndrome can cause ID. The new approach “reverse genetics,” where the genotype is tested before the full clinical phenotype is developed, is a successful approach in view of the growing microdeletion/microduplications syndromes [2].

FGS1 was initially described by Opitz et al. in 1974 as a rare X-linked disorder [3]. It is a mental retardation syndrome characterized by congenital hypotonia, imperforate anus, constipation, partial agenesis of the corpus callosum, behavioral disturbances, and dysmorphic features such as absolute or relative macrocephaly, tall forehead, down-slanted palpebral fissures, small and simple ears, broad thumbs and halluces [4]. FGS1 is mapped to Xq13 in a gene called MED12, which appears to be the most common cause of the disorder. The disease’s prevalence is rare worldwide, and it is not published in Saudi Arabia. This syndrome can be diagnosed clinically by typical features and confirmed by genetic testing. In this communication, we described the first published unbalanced translocation (13;18) (q34,q23) and de novo microdeletion 6q25 syndrome; there is a history of the paternal use of methotrexate during the conception of those two boys.

## Case presentation

Patient 1

**Figure 1:**
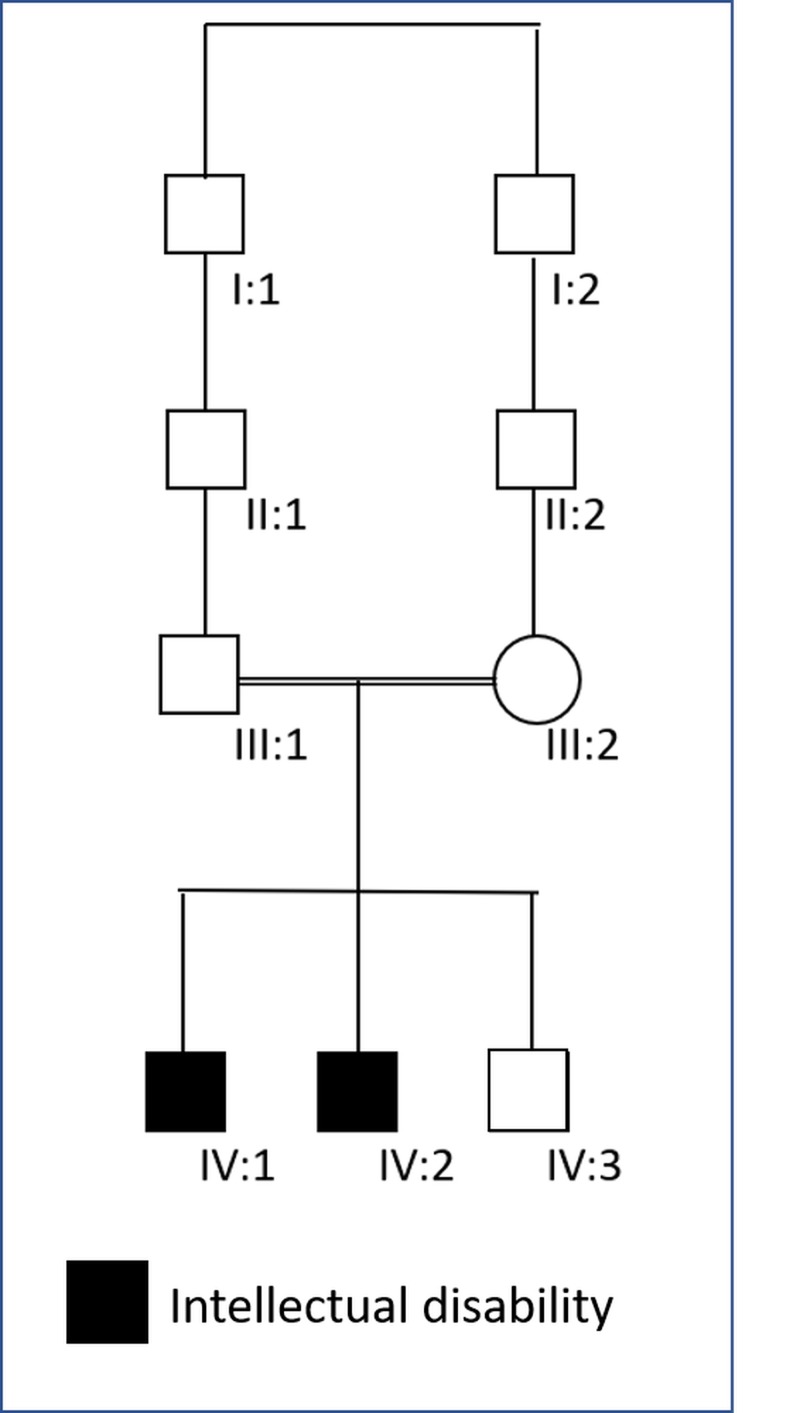
Family pedigree, showing two affected kids with Intellectual disability Patient 1 is (IV:1); Patient 2 is (IV:2)

**Figure 2:**
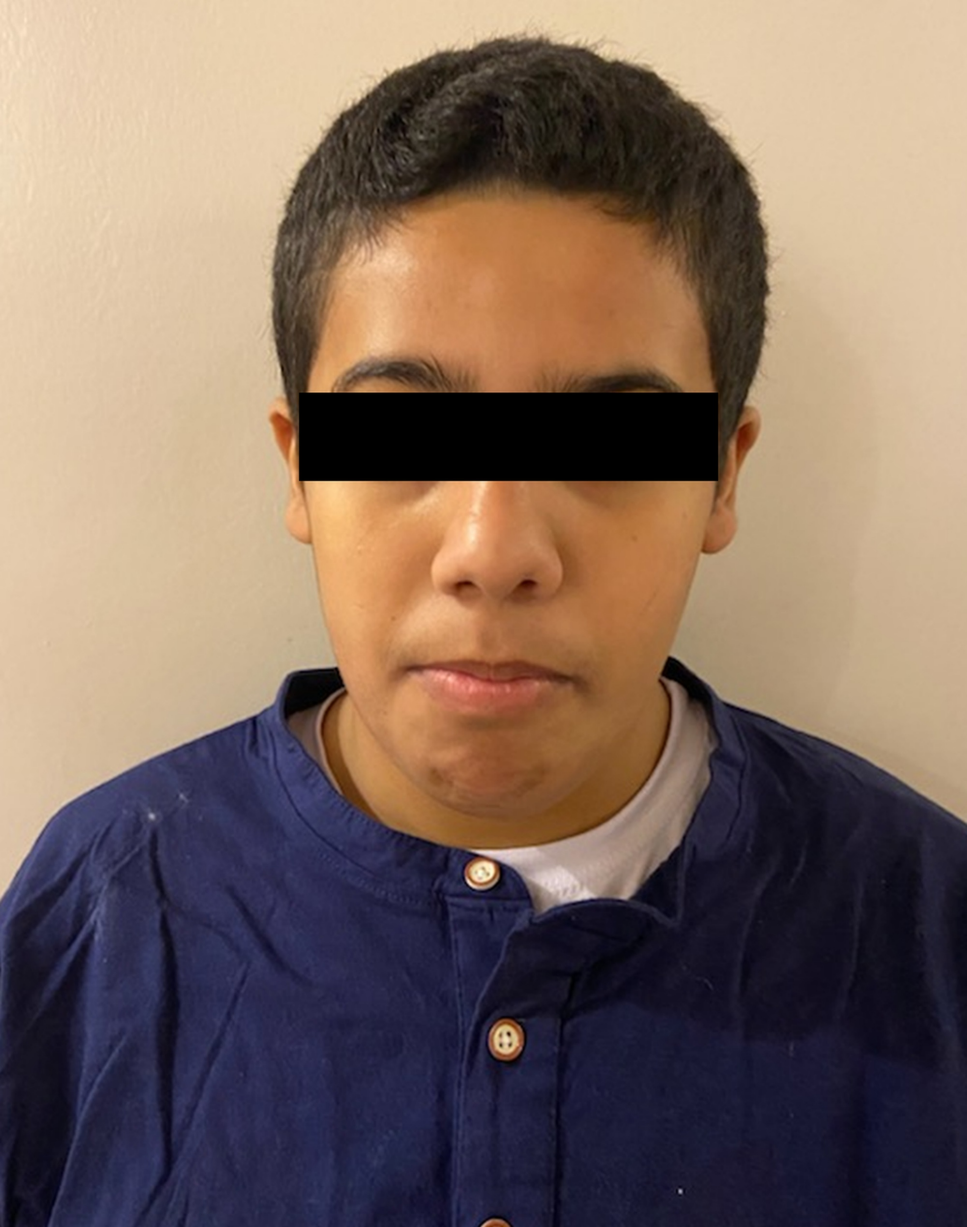
Patient 1 showed micrognathia and a long philtrum

A 13-year-old Saudi boy, a product of a full-term, normal, spontaneous vertex delivery after an uneventful pregnancy, presented. He had normal development until the age of 18 months when he developed his first tonic-clonic convulsion, followed by postictal sleeping. His parents noted that he started to have a delay in gaining more gross motor skills, had delayed speech, and became hyperactive. At the age of four years, he was diagnosed to have attention deficit hyperactivity disorder (ADHD) and showed poor school performance. At the same age, the parents noticed his increase in appetite and weight. He had recurrent otitis media that required tube insertion at the age of six years. He was found to have hypermetropia at 10 years. Family history showed that parents are third cousins and had one affected brother with a similar presentation and one normal brother (Figure 1). His father is known to have rheumatoid arthritis on methotrexate. On physical examination, he was an obese child, with weight 94 kg (more than the 90th percentile), height 140 cm (below the 5th percentile), his body mass index (BMI) was 48 kg/m^2^. The patient has a high forehead and micrognathia and a long philtrum (Figure 2). The patient underwent extensive biochemical and metabolic workup that included tandem mass spectrometry (MS), urine gas chromatography-mass spectrometry (GCMS), lactate, creatine phosphokinase (CPK), plasma amino acid, and liver function test; all were inconclusive. MRI brain showed corpus callosum agenesis. Cardiac echo showed a large atrial septal defect of the secundum type closed spontaneously. Karyotype showed normal 46,XY; array comparative genomic hybridization (CGH) showed unbalanced (13;18) (q34,q23) translocation with heterozygous terminal deletion of about 2.5 mega based in 13q34 (111,553,901-114,124,062) and terminal duplication about 2.5 mega based on 18q23 (73,516,340-76,111,164). Whole exome sequence showed he had a variant of uncertain significance in MED12(NM_005120.2):c.3797G>A (p.R1266H) in exon 27. Diagnostic confirmatory of the MED 12 gene analysis was done, which showed the same mutation in a hemizygous form in the mother and one affected brother and another normal brother (see family pedigree).

Karyotype and array CGH for the parents were negative. The patient continued to have the behavioral problem of eating disorder and hyperactivity, affability, and excessive talkativeness, along with socially oriented, attention-seeking behaviors.

Patient 2

**Figure 3:**
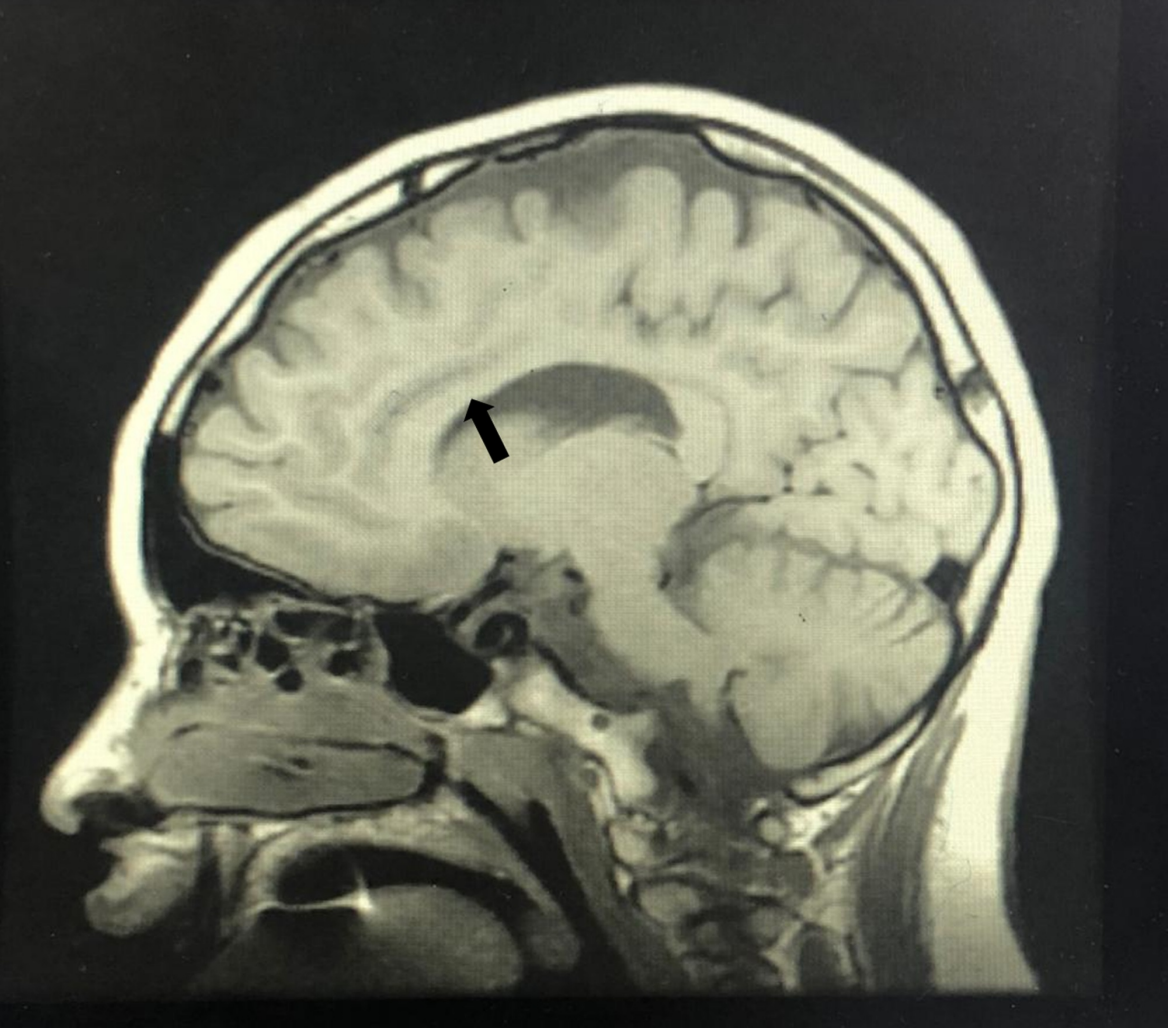
Agenesis corpus callosum; sagittal T1-weighted MRI of the brain shows partial agenesis of the corpus callosum (black arrow)

**Figure 4:**
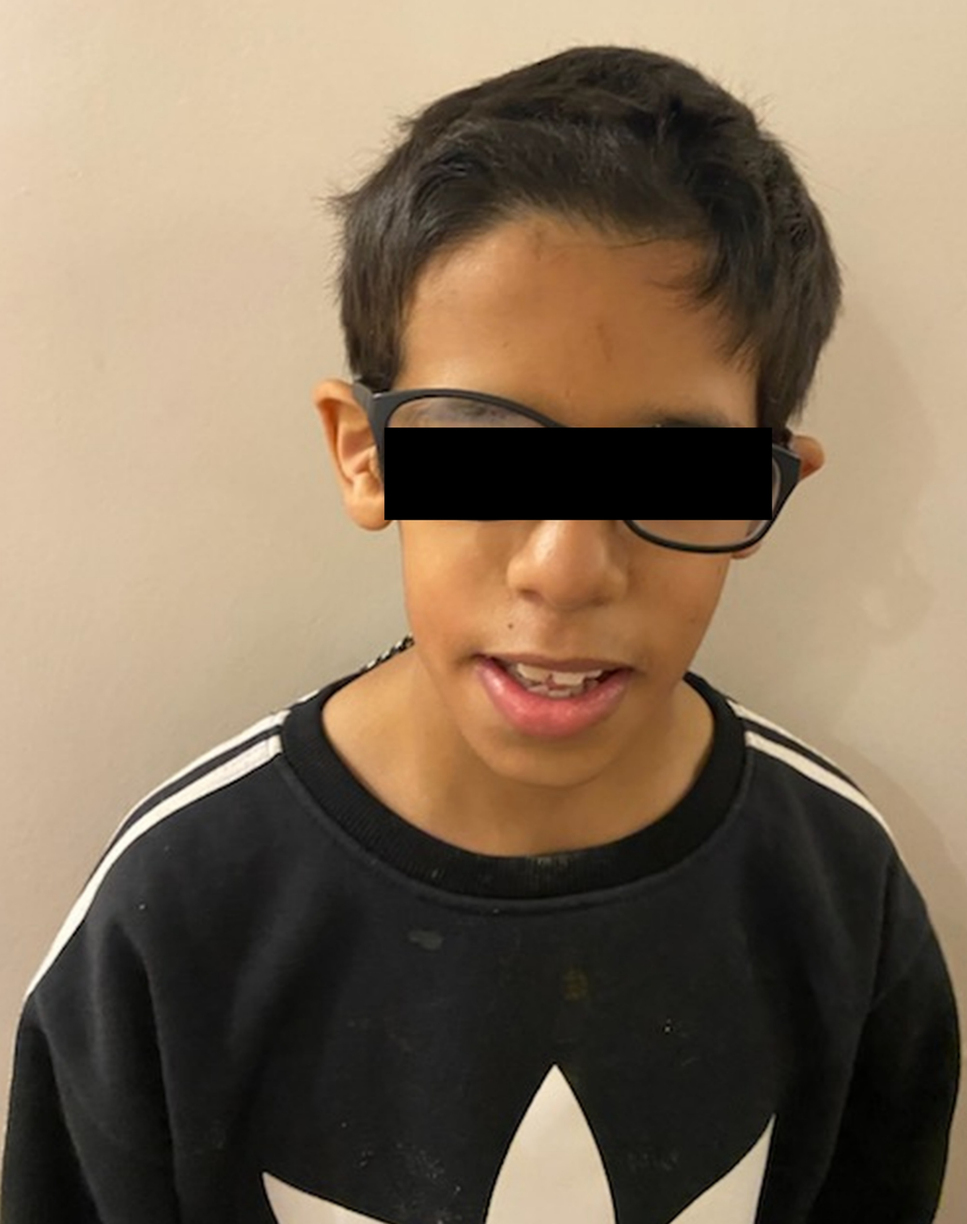
Patient 2 showing large nose and prominent forehead

The second brother is an 11-year-old boy, a product of a full-term, spontaneous vertex delivery, with a birth weight of 3.2 kg. At day two of life, he developed a seizure, which was tonic and become atonic with postictal sleep. He had hyperactivity with attention deficit and developmental delay - he sat at 16 months and started to walk with support at age two years, had also delayed speech: at 2 ½ years, he said only two words. On examination, the patient had mild dysmorphic features, his head circumference was at the 50th percentile and length at the 50th percentile. He has short palpebral fissure, hypertelorism, epicanthal folds, bitemporal narrow, post-posterior sloping of hair, low set ears with mild micrognathia, high arch of the palate, clinodactyly, and nystagmus. The patient underwent extensive biochemical and metabolic workup that includes a tandem MS, urine GCMS, lactate, CPK, plasma amino acid, and liver function test; all were inconclusive. MRI brain showed agenesis of the corpus callosum (Figure 3). Figure 4 shows Patient 2.

Karyotype was 46,XY normal male, array CGH showed pathogenic reduced dosage of genomic material in terms of a heterozygous deletion (approx. 9.9Mb) on the long arm of chromosome 6 (heterozygous deletion in 6q25.3-q27 (160,849,200-170,748,862)) with causative relevance for the phenotype. Sanger confirmed a pathogenic variant in MED12(NM_005120.2):c.3797G>A (p.R1266H) in the exon 27 gene. He did not develop obesity like his brother. There is a history of paternal methotrexate ingestion before his conception. The third brother, however, had normal development, normal CGH array, and carried the same MED12 variant; the father was off methotrexate for more than five months during the conception of this third boy.

## Discussion

Usually, 18q23 duplication varies from a rare severe phenotype consisting of complex cortical malformations, facial anomalies, and heart structural defects, to common clinical features with patients with full trisomy 18 (agenesis of the corpus callosum, cerebellar hypoplasia, and heart septal defects [5]. Terminal microdeletions of 13q34 lead to developmental delay and/or intellectual disability in the vast majority of affected individuals. Facial dysmorphism and microcephaly were reported in about half of the overall reported cases, convulsions were noted in one-fifth of the patients, while heart anomalies, short stature, and hypotonia each involved about 10%-30% of the cases [6]. Obesity can also be part of the 13q deletion syndrome but it’s not a specific feature and frequently results from intellectual disability [7]. This boy suffers from morbid obesity, which was referred to Pediatric Surgery for considering surgical management.

Interstitial and terminal deletions of 6q25 are rare and are associated with developmental delays, craniofacial anomalies, hypotonia, seizures, and mild to moderate intellectual disability. Affected patients may have microcephaly, motor disorders, speech disorder, ataxic cerebral palsy, optic nerve dysplasia, and agenesis of the corpus callosum as well. The second boy had some of those features, however, he does not have eye pathology [8].

FG syndrome is a rare, X-linked, multiple congenital anomaly-cognitive impairment disorder, characterized by intellectual disability and hyperactivity, with a short attention span and hypotonia. It also tends to have a characteristic facial appearance, including underdeveloped small ears, prominent forehead, upswept frontal hairline, and large head size as compared to body size [9]. Patients may have other health problems, including seizures, heart defects, undescended testes, and inguinal hernia. Those three brothers inherited this variant from an asymptomatic mother, however, the first boy had morbid obesity, which was reported in the 13q deletion. The second brother had the typical features of congenital hypotonia and delayed development, with no obesity; moreover, the youngest brother had only mild hypotonia at infancy that improved later. The variant MED12 (p.R1266H) was reported as a variant of uncertain significance; moreover, it did not segregate well in the family, as all boys were hemizygous, even the normal one; the mother was a carrier while the father carried the wild type variant [10].

Methotrexate (MTX) has become established as the most commonly used disease-modifying anti-rheumatic drug (DMARD) in the treatment of rheumatoid arthritis (RA) and is widely used in other inflammatory conditions [11]. The safety and potential hazards to the fetus when the father is exposed to methotrexate prior to conception is unclear [12]. The current recommendations to avoid paternal MTX use before conception do not appear to be supported by evidence and paternal treatment with MTX could be continued when planning a pregnancy [12]. There has been discussion in the literature regarding surveillance with amniocentesis, and there is no evidence to recommend primary invasive diagnostics, as the risk associated with the procedure seems to outweigh the benefits [13]. In our case, the father was on methotrexate during the pregnancy of the first two brothers; however, he was off medication during the conception of the third one.

## Conclusions

We report two brothers with intellectual disabilities and a severe speech delay. The whole-exome sequence showed a hemizygous, likely benign, variant in MED12 in all of them, inherited from the asymptomatic mother. They have another chromosomal microdeletion-microduplication anomaly, which is de novo in nature. Paternal use of chemotherapy during the conception has a controversial effect. The physician should look for another disease in the same family if the symptoms were not explained fully by the genetic disorder.
